# Supplementary material for: Distinct neural mechanisms underlie subjective and objective recollection and guide memory-based decision making
Source: eLife. 2021 Mar 9;10:e62520. doi: 10.7554/eLife.62520 (PMC7943194; doi:10.7554/eLife.62520)
Supplement: Transparent reporting form [file elife-62520-transrepform.docx]

***eLife’s* transparent reporting form**

We encourage authors to provide detailed information *within their submission* to facilitate the interpretation and replication of experiments. Authors can upload supporting documentation to indicate the use of appropriate reporting guidelines for health-related research (see [EQUATOR Network](http://www.equator-network.org/%20)), life science research (see the [BioSharing Information Resource](https://biosharing.org/)), or the [ARRIVE guidelines](http://www.plosbiology.org/article/info:doi/10.1371/journal.pbio.1000412) for reporting work involving animal research. Where applicable, authors should refer to any relevant reporting standards documents in this form.

If you have any questions, please consult our Journal Policies and/or contact us: [editorial@elifesciences.org](mailto:editorial@elifesciences.org).

**Sample-size estimation**

- You should state whether an appropriate sample size was computed when the study was being designed
- You should state the statistical method of sample size computation and any required assumptions
- If no explicit power analysis was used, you should describe how you decided what sample (replicate) size (number) to use

Please outline where this information can be found within the submission (e.g., sections or figure legends), or explain why this information doesn’t apply to your submission:

We did not conduct a formal power analysis, but we selected the sample size for this experiment (N=30) based on the moderate effect sizes for the dissociation between objective and subjective memory measures observed in each of four behavioral experiments included in Hembacher and Ghetti (2016). The current sample size corresponds to the largest employed in one of those behavioral experiments. This information can be found on pp. 25-26 of the submitted manuscript.

**Replicates**

- You should report how often each experiment was performed
- You should include a definition of biological versus technical replication
- The data obtained should be provided and sufficient information should be provided to indicate the number of independent biological and/or technical replicates
- If you encountered any outliers, you should describe how these were handled
- Criteria for exclusion/inclusion of data should be clearly stated
- High-throughput sequence data should be uploaded before submission, with a private link for reviewers provided (these are available from both GEO and ArrayExpress)

Please outline where this information can be found within the submission (e.g., sections or figure legends), or explain why this information doesn’t apply to your submission:

We initially developed the paradigm for behavioral research and the dissociation between objective and subjective memory measures reported in the manuscript has been found and replicated in adult samples across four experiments (Hembacher & Ghetti, 2016). This information is reported in the Introduction (on pp. 4-6).

**Statistical reporting**

- Statistical analysis methods should be described and justified
- Raw data should be presented in figures whenever informative to do so (typically when N per group is less than 10)
- For each experiment, you should identify the statistical tests used, exact values of N, definitions of center, methods of multiple test correction, and dispersion and precision measures (e.g., mean, median, SD, SEM, confidence intervals; and, for the major substantive results, a measure of effect size (e.g., Pearson's r, Cohen's d)
- Report exact p-values wherever possible alongside the summary statistics and 95% confidence intervals. These should be reported for all key questions and not only when the p-value is less than 0.05.

Please outline where this information can be found within the submission (e.g., sections or figure legends), or explain why this information doesn’t apply to your submission:

Statistical analysis methods are described in detail on p. 28 (behavioral analyses) and p. 29 (neuroimaging analyses) of the manuscript. The results (p. 8 -17) include detailed description of the specific statistical tests we used along with partial eta squared as a measure of effect size for all reported effects. Additionally, we report exact p-values for all tests, with the exception of those estimated at p <.001. Raincloud plots of all main results including raw data points, boxplots, means, standard errors and distributions are provided in Figures 2 – 4. Whole-brain images were thresholded at Z > 3.1, cluster-corrected at p < .05. Subsequent regions of interest (ROI) analyses were corrected for multiple comparisons within a contrast using a false discovery rate (FDR) correction. This information is provided in methods (p. 30).

(For large datasets, or papers with a very large number of statistical tests, you may upload a single table file with tests, Ns, etc., with reference to sections in the manuscript.)

**Group allocation**

- Indicate how samples were allocated into experimental groups (in the case of clinical studies, please specify allocation to treatment method); if randomization was used, please also state if restricted randomization was applied
- Indicate if masking was used during group allocation, data collection and/or data analysis

Please outline where this information can be found within the submission (e.g., sections or figure legends), or explain why this information doesn’t apply to your submission:

The present experimental paradigm included a within-subject manipulation. Thus, participants were not assigned to different groups. Participant assignment to ID was random. Assignments of stimuli to each within-subject condition was randomized across participants. The positions of response buttons during the decision phase (Select versus Discard) was counterbalanced across participants via a random assignment of position to ID.

**Additional data files (“source data”)**

- We encourage you to upload relevant additional data files, such as numerical data that are represented as a graph in a figure, or as a summary table
- Where provided, these should be in the most useful format, and they can be uploaded as “Source data” files linked to a main figure or table
- Include model definition files including the full list of parameters used
- Include code used for data analysis (e.g., R, MatLab)
- Avoid stating that data files are “available upon request”

Please indicate the figures or tables for which source data files have been provided:

Numerical data related to all figures is provided in corresponding source data files accompanying this submission.
